# Unusual Presentation of Parieto-Visceral Peritoneal Band Obstruction in Adults: The Role of Congenital Peritoneal Bands

**DOI:** 10.7759/cureus.92809

**Published:** 2025-09-20

**Authors:** Vinay Sharma, Padamjeet Panchal, C. S Rameshbabu

**Affiliations:** 1 Department of Anatomy, Muzaffarnagar Medical College, Muzaffarnagar, IND; 2 Department of Anatomy, All India Institute of Medical Sciences, Patna, Patna, IND

**Keywords:** anterior abdominal wall, cadaveric dissection, congenital anomaly, embryological development, greater omentum, parieto-visceral fold, peritoneal band, small bowel obstruction, stomach compression, transverse colon

## Abstract

Peritoneal bands are rare congenital remnants of mesenteric folds that may persist due to incomplete embryological regression. While they are a recognized cause of small bowel obstruction in children, their occurrence in adults is uncommon and often incidental.

During routine abdominal dissection of a 42-year-old female cadaver, an atypical parieto-visceral peritoneal band was identified. The band originated from the anterior abdominal wall, approximately two inches above the umbilicus near the apex of the falciform ligament, and coursed posteriorly. It exerted a constrictive effect on the distal stomach at the greater curvature just proximal to the pylorus. The fold bifurcated before termination: one branch blending with the greater omentum and the other attaching to the transverse colon. Fibrous near its origin and fatty distally, the band also contained small vascular twigs. No other anatomical variations or pathological changes were observed. This case illustrates a rare congenital peritoneal band producing gastric constriction without intestinal obstruction. Awareness of such variants is important for surgeons and radiologists to avoid diagnostic confusion or inadvertent intraoperative injury.

## Introduction

Intricate development of the mesentery and gastrointestinal tract during embryonic growth creates a foundation for the formation of several diverse membranes, veils, and folds that are collectively referred to as peritoneal bands [1]. Small bowel obstruction due to peritoneal bands is commonly associated with pediatric age. In adults, 60% of small bowel obstructions are caused by adhesions, most commonly postoperative. Peritoneal bands frequently extend from the cecum toward the upper quadrant on the right side of the retroperitoneum, where they may entrap the transverse and descending segments of the duodenum. Such anomalous folds exert varying degrees of constriction, almost invariably culminating in obstruction of the small bowel. Notably, congenital peritoneal bands have been implicated in approximately 3% of all cases of intestinal obstruction, underscoring their clinical relevance despite their relative rarity [2]. Anomalous peritoneal bands may occur without prior history of surgery or intraperitoneal inflammation. Sometimes, the developmental basis of aberrant congenital peritoneal bands has not been clearly established, with several hypotheses proposed regarding their origin. These aberrant bands originate during embryogenesis, representing persistent vestiges of the ventral mesentery that fail to undergo the expected process of regression [3,4]. Disturbances in embryological development of the gastrointestinal tract may yield outcomes that span a wide spectrum, from severe, clinically significant manifestations to entirely silent anomalies. Among these, congenital malrotation is of particular concern, with approximately 80% of affected neonates exhibiting clinical features within the first month of life. During the 28th day of intrauterine life, when the intestines achieve their definitive anatomical position, a developmental anomaly in the mesentery may result in aberrant bands. At this juncture, the mesenteries are normally apposed to the evolving dorsal abdominal wall, where they fuse with the parietal peritoneum and ultimately undergo regression [4]. In the present case, we report an unusual observation in which the stomach was compressed by a parieto-visceral peritoneal band.

Anomalous peritoneal bands may occur without prior history of surgery or intraperitoneal inflammation, as in the present case, and can exert compressive effects or cause obstruction. The band observed was a single linear structure with no evidence of prior insult. Its etiology remains uncertain.

## Case presentation

**Figure 1:**
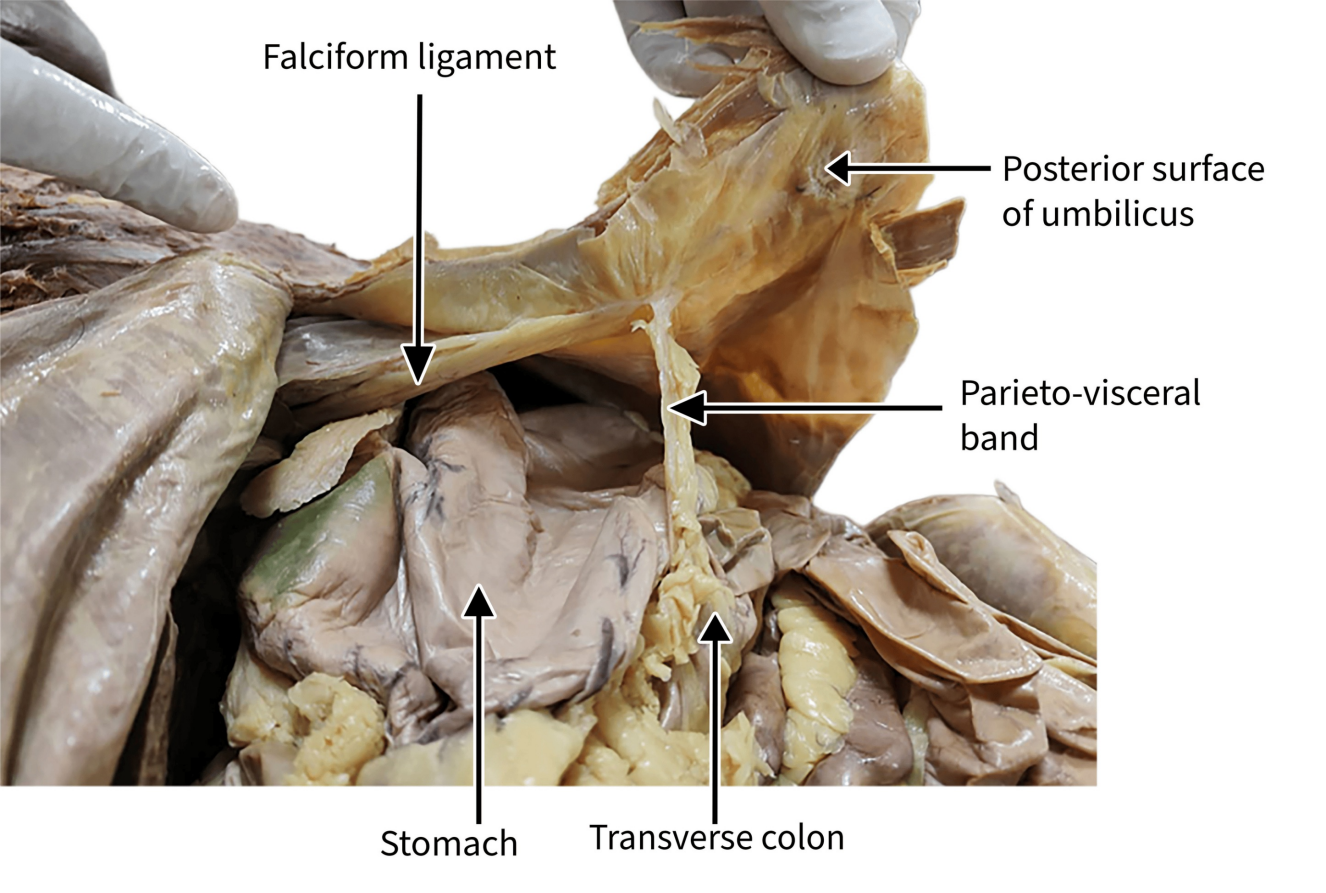
Cadaveric dissection demonstrating an anomalous parieto-visceral peritoneal band.

During routine cadaveric dissection of the abdominal region, an atypical peritoneal band was observed in a 42-year-old female cadaver. The band exerted a conspicuous constrictive effect on the distal stomach along the greater curvature (GC), immediately proximal to the pyloric region. Careful anatomical delineation demonstrated that this anomalous fold originated from the anterior abdominal wall, coursed toward the greater omentum, and continued to form an additional attachment with the anterior surface of the transverse colon (Figure 1).

**Figure 2:**
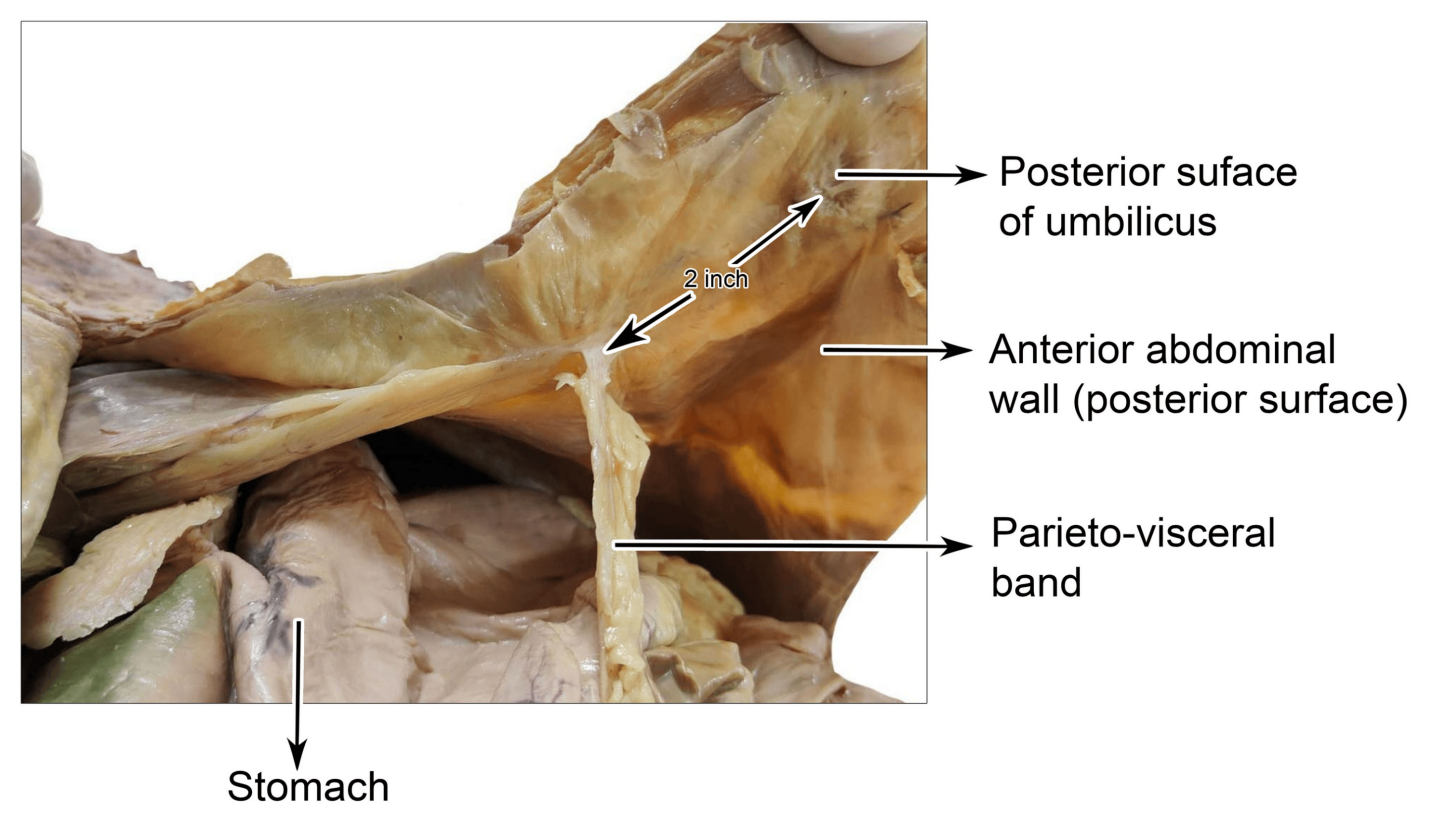
Cadaveric dissection showing the parieto-visceral peritoneal band arising from the posterior surface of the anterior abdominal wall, approximately two inches above the umbilicus.

One end of the band was attached in the midline approximately two inches above the umbilicus, immediately near the attachment of the apex of the falciform ligament. It must be noted that the apex of the falciform ligament was not attached to the umbilicus (Figure 2).

**Figure 3:**
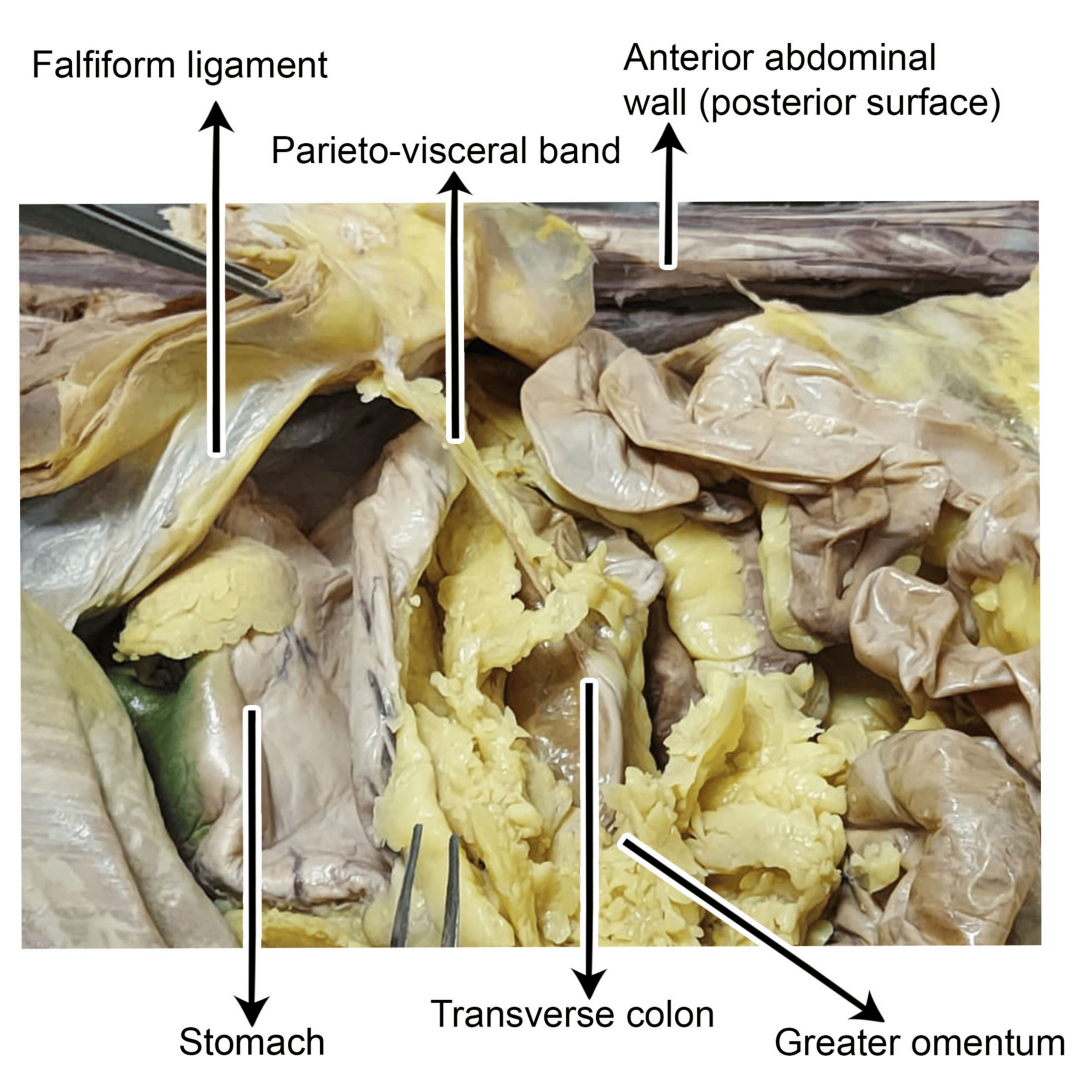
Dissection of the abdominal cavity showing the parieto-visceral peritoneal band extending from the posterior surface of the anterior abdominal wall near the falciform ligament, coursing toward the greater omentum, stomach, and transverse colon.

On gross examination, the band near its parietal attachment was more fibrous in nature (Figure 3).

**Figure 4:**
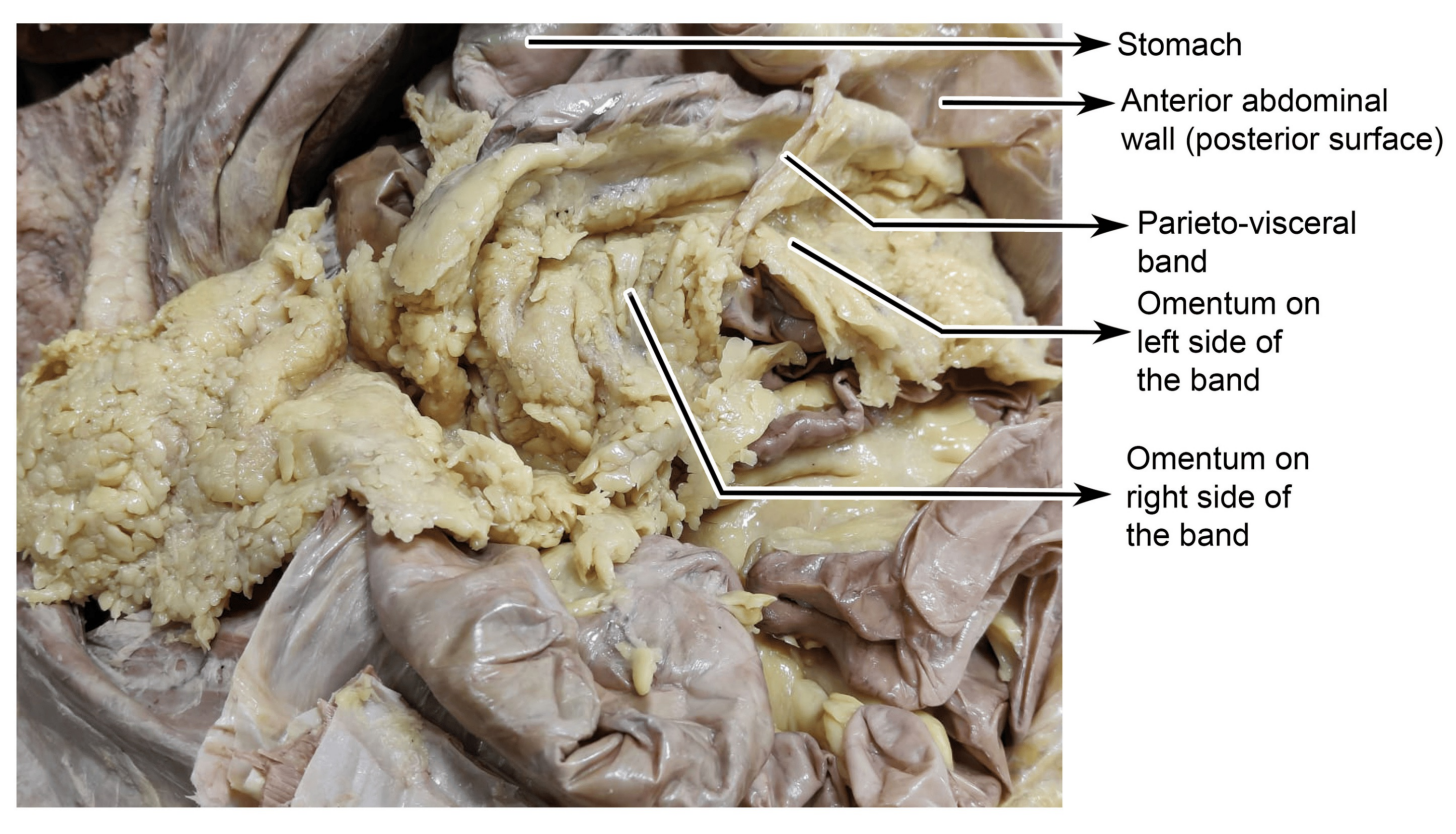
Cadaveric dissection showing the parieto-visceral peritoneal band in relation to greater omental.

On gross examination, as the band coursed superiorly and posteriorly, it appeared thickened and was laden with adipose tissue. During its course, it compressed the ventral surface near the greater curvature of the stomach. While running posteriorly, it was observed that some parts of the greater omentum were staggered, looping over the band. This might partially restrict the distension of the stomach. In addition, a relatively larger portion of the greater omentum was displaced toward the right side, with only a smaller portion extending to the left, suggestive of a restrictive influence exerted by the parieto-visceral band (Figure 4).

**Figure 5:**
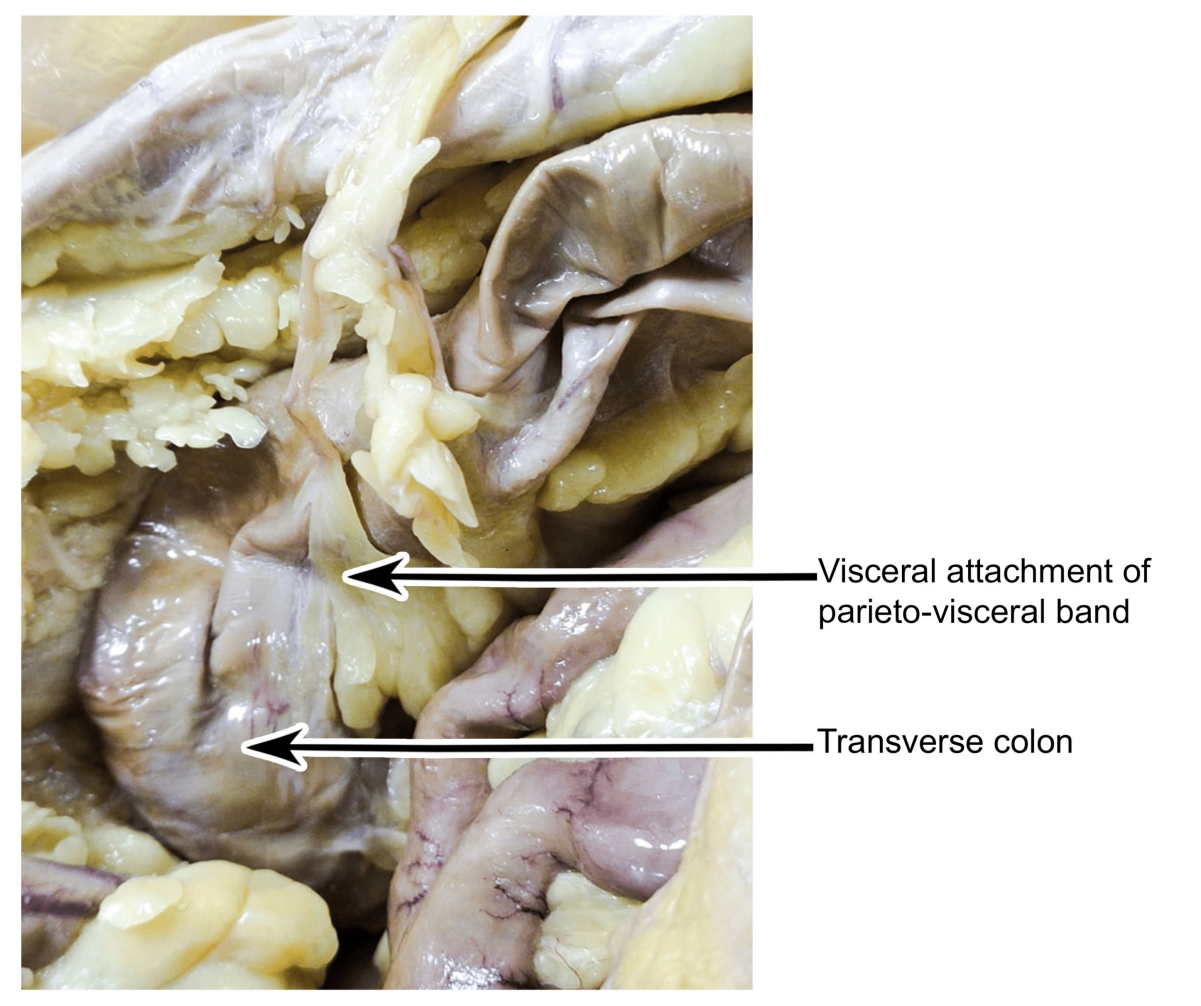
Visceral attachment of the parieto-visceral peritoneal band to the transverse colon as observed during cadaveric dissection.

Before termination, it bifurcates into two parts. One part becomes flattened and expanded and then blended with the greater omentum near the greater curvature of the stomach, and the other part is narrowed down and tapered, reaching deeper, just crossing the midline to gain attachment to the transverse colon. Some twigs of vessels were observed in the middle and visceral ends of the band (Figure 5).

This abnormal fibrous attachment restricted the mobility of the greater omentum and was partially constrictive on stomach distension. Further meticulous exploration of the abdominal cavity revealed no additional gross pathological alterations or anatomical variations. The remaining visceral and peritoneal structures exhibited the expected topographical relationships and morphological features. The abdomen was non-distended with no sign of peritoneal irritation. There were no distended loops of the small bowel.

A detailed visualization of both the anterior abdominal wall and the visceral relationships, highlighting the precise course and attachment of the peritoneal band, has been clearly demonstrated in the cadaveric dissection (Video 1).

**Video 1:** Cadaveric demonstration of a parieto–visceral peritoneal band.

## Discussion

A parieto-visceral band constitutes an uncommon, anomalous congenital peritoneal structure, defined by the presence of an aberrant fibrous or membranous adhesion that establishes an abnormal connection between a visceral organ and the abdominal wall lined by the parietal peritoneum. While the majority of peritoneal folds arise during embryogenesis as structured suspensory laminae that secure the abdominal viscera in their definitive anatomical positions, parieto-visceral bands represent an uncommon deviation from this normative process. These anomalous adhesions, although infrequently encountered, possess substantial clinical significance owing to their propensity to precipitate critical complications.

By the 26th day of intrauterine life, during gastric rotation, the ventral and dorsal mesenteries divide the peritoneal cavity into the right and left compartments. At this stage, congenital bands may develop between the ventral and dorsal mesogastrium as a result of embryonic adhesions [3,5]. A persistent ventral mesentery can give rise to peritoneal bands, which account for approximately 3% of patients detected with small bowel obstruction. While the dorsal mesentery persists in the posterior abdominal region and subsequently fuses with the parietal peritoneum, the ventral mesentery regresses normally as the intestine reaches its definitive position. These anomalous bands may also coexist with other congenital anomalies arising during intestinal rotation, such as duodenal atresia, enteric duplications, gut malrotation, and, in rare instances, superior mesenteric artery syndrome [3,6].

The midgut undergoes a characteristic 270° counterclockwise rotation during the 10th to 11th weeks of gestation, while the remaining gut is held in place by the mesentery, a double-layered serous membrane that differentiates into dorsal and ventral components. Although most primitive mesenteries disappear by the end of fetal life, persistent remnants can form anomalous peritoneal bands, which are most frequently identified near the duodenum, duodenojejunal flexure, jejunum, terminal ileum, cecum, and different segments of the colon [1]. Errors in this rotation process may permit abnormal fixation of the mesentery and intestine, predisposing to the formation of peritoneal bands that can cause internal herniation, mechanical obstruction, or volvulus [3].

Occasionally, an atypical configuration resembling a developmental remnant of the cecal mesentery has been identified on the left side, presenting as a vascularized peritoneal fold coursing toward and extending into the iliac fossa. This anomalous attachment was found to tether the vermiform appendix, which occupied an unusual midline position, lying anterior to the ileal loops. A vascular anastomotic vessel within the band was noted connecting the left colic artery and the ileocolic artery [7]. Finech et al. documented a rare instance in which the stomach was strangulated by an anomalous peritoneal band connecting the intestinal mesentery to the diaphragm [8]. A similar instance of an abnormal band extending from the lesser to the greater omentum compressed the stomach [9]. Sozen et al. reported a case of intestinal obstruction caused by an anomalous peritoneal band from the antimesenteric border of the terminal ileum to the mesoappendix that contained arteries, veins, and nerve roots [2]. An instance has also been documented in which a congenital band obstructed the proximal jejunum [10]. A rare occurrence of an abnormal cystogastrocolic peritoneal fold has also been documented [11]. Additionally, a cadaveric case has been described in which the greater omentum was found to be attached to the falciform ligament [12].

Owing to the inadequate number of external indications that can be observed during the clinical evaluation of patients with peritoneal abnormalities, diagnosing these anomalies is challenging. The best first-line imaging is plain radiography, which can show multiple air-fluid levels and dilated bowel loops. Barium studies are considered the gold standard for identifying obstructions, as they can reveal blockages within the small intestine and detect displaced intestines within the abdomen, potentially indicating the presence of peritoneal band abnormalities. Contrast CT may allow perfusion anomalies to be visualized more clearly and can detect a band in suspected obstruction [13].

Awareness of anomalous peritoneal bands and their vascularity is of paramount importance to both surgeons and radiologists. Such aberrant folds, along with the recesses they create, may predispose patients to pathological conditions and can also provide potential routes for the dissemination of malignant diseases [12].

## Conclusions

Congenital peritoneal bands are rare developmental remnants that typically remain silent but may assume clinical importance when they compress or obstruct gastrointestinal structures. Although most commonly implicated in pediatric small bowel obstruction, their occurrence in adults is rare and often incidental. The present cadaveric case highlights an atypical parieto-visceral peritoneal band producing gastric constriction without evidence of obstruction, underscoring the wide spectrum of possible presentations. Awareness of such anomalous folds and their variable vascular content is crucial for anatomists, surgeons, and radiologists alike, as failure to recognize them may lead to diagnostic confusion or inadvertent intraoperative injury.
